# Investigations on a Novel Inductive Concept Frequency Technique for the Grading of Oil Palm Fresh Fruit Bunches

**DOI:** 10.3390/s130202254

**Published:** 2013-02-07

**Authors:** Noor Hasmiza Harun, Norhisam Misron, Roslina Mohd Sidek, Ishak Aris, Desa Ahmad, Hiroyuki Wakiwaka, Kunihisa Tashiro

**Affiliations:** 1 Faculty of Engineering, Universiti Putra Malaysia, Serdang, Selangor 43400, Malaysia; E-Mails: noorhasmiza@bmi.unikl.edu.my (N.H.H.); roslina@eng.upm.edu.my (R.M.S.); ishak@eng.upm.edu.my (I.A); desa@eng.upm.edu.my (D.A.); 2 Insitute of Advanced Technology (ITMA), Universiti Putra Malaysia, Serdang, Selangor 43400, Malaysia; 3 Department of Electrical & Electronic Engineering, Faculty of Engineering, Shinshu University, Wakasato 4-17-1, Nagano 380-8553, Japan; E-Mails: wakiwak@shinshu-u.ac.jp (H.W.); tashiro@shinshu-u.ac.jp (K.T.)

**Keywords:** inductive, resonant frequency, air coil, oil palm, frequency characteristics, maturity classification, inductive concepts

## Abstract

From the Malaysian harvester's perspective, the determination of the ripeness of the oil palm (FFB) is a critical factor to maximize palm oil production. A preliminary study of a novel oil palm fruit sensor to detect the maturity of oil palm fruit bunches is presented. To optimize the functionality of the sensor, the frequency characteristics of air coils of various diameters are investigated to determine their inductance and resonant characteristics. Sixteen samples from two categories, namely ripe oil palm fruitlets and unripe oil palm fruitlets, are tested from 100 Hz up to 100 MHz frequency. The results showed the inductance and resonant characteristics of the air coil sensors display significant changes among the samples of each category. The investigations on the frequency characteristics of the sensor air coils are studied to observe the effect of variations in the coil diameter. The effect of coil diameter yields a significant 0.02643 MHz difference between unripe samples to air and 0.01084 MHz for ripe samples to air. The designed sensor exhibits significant potential in determining the maturity of oil palm fruits.

## 1. Introduction

In agricultural applications, the quality of the harvested product (fruit) is the major factor that contributes to the profit. Generally, the quality of fruit is categorized based on the texture, shape and color [1]. In case of the oil production from oil palm fresh fruit bunches (FFBs), the quality of oil produced is also an important factor for the harvester. Therefore, it is crucial to harvest the oil palm FFBs at the correct time to maximize the production of palm oil.

Malaysia is one of the largest exporters of palm oil in the World, contributing 3.2% to the country's real gross domestic product [1]. Currently, Malaysian harvesters use a human expert grading approach to inspect the maturity of bunches and classify them for harvesting. Factors such the color of the mesocarp (surface of the fruitlet) and also the number of loose fruits from bunches are used to refer them for harvesting [2]. This method is monotonous and often leads to bunch misjudgment leading to the compromises in the production of the palm oil and causing considerable profit losses [2,3]. With the prevailing issues due to human grading nowadays the need for an automated method to detect the maturity of the oil palm FFBs is drawing considerble interest among the researchers in Malaysia.

Various automated fruit grading systems have been proposed and tested for practical usage over the past few years. The most popular method is the use of color vision systems wherein an advanced digital camera, a set of personal computers and a trained operator are required [4–7]. This method requires supporting equipment and is not suitable for on-site testing. The system is also sometimes accompanied by an artificial intelligence system to classify the oil palm fresh fruit bunches [8,9]. Neural networks and fuzzy regression models are the most competent methods used by researchers for the classification [10,11]. It is known that the method requires a complicated algorithm and precise image collection for the recognition stages.

Oil palm fresh fruit bunch ripeness assessment using RGB space wherein the spectral analysis based on different wavelength of red, green and blue color of the image is another method used by researchers in this field [12,13]. As the method totally depends to the color quality of the image, the feature extraction plays an important role in this method. The method implied a successful classification of the ripe category within the bunch with average value of red component. However, it is unable to differentiate the red component for unripe and under ripe categories [14]. Additionally, this method requires human graders to select the samples for the image acquisition procedure and the classification of sample has to be performed indoors [14,15]. Another method applying the RGB space method is known as the photogrammetric grading system, which requires regular imaging technology components such as image acquisition, image pre-processing and image segmentation [14,15]. This system is also only suitable for indoor testing where the support equipment required for capturing the images of oil palm fresh fruit bunches can be deployed.

Use of the moisture content of the oil palm, specifically the mesocarp component that contains the oil, is another major aspect in the automated grading system research field [16]. The ripening process leads to a minimum moisture content in the mesocarp [1]. This moisture content of the mesocarp highly affects the surface color and the weight of the oil palm fruit that can be investigated using various types of microwave moisture sensors [16]. For instance, microstrip and coplanar sensors are used to estimate the moisture content in the oil palm fruit based on attenuation measurements. However the procedure involved in this process is quite complicated and time consuming as it requires difficult preparation of sample where the fresh mesocarp should be mashed into a semi solid form. The drawback of the microstrip and coplanar sensors is addressed with the usage of open ended coaxial cable as the moisture sensor [16]. The fresh sample of mesocarp is sliced partially to allow a good contact between the open ended coaxial cable with the surface of the mesocarp. Unfortunately, this method requires a very high frequency range of up to 5 GHz and involves tedious sample preparation steps [16].

Magnetic Resonance Imaging (MRI) and bulk Nuclear Magnetic Resonance (NMR) are other methods proposed by researchers to monitor the development and ripeness of FFBs [17]. FFB samples are harvested at 4, 12, 16 and 21 weeks after anthesis (WAA). Both types of equipment were used to measure the continuous change in the spin-spin relaxation times (T_2_-values) of the protons of the water and lipids for the development of a ripening tracking process for FFBs. This study demonstrated significant changes between oil and moisture content in FFBs based on the differences in their spin-spin relaxation times. The main drawback of this method is the usage of complicated and expensive equipment that requires skilled personnel to operate it. It is suitable for indoors and laboratory experimental testing of FFBs. Another imaging method involved nondestructive near infrared (NIR) spectroscopy for the determination of palm oil in oil palm fruits. The oil palm fruits were scanned by two NIR spectrometers with different modes. The chemical content of the palm oil was analysed using partial least squares regression (PLSR) models [18]. Apart from the usage of the imaging technology, a capacitive concept was proposed too [19]. The concept applied was to measure the dielectric properties of a mashed mesocarp. Measurements involved from low frequency range (10^−2^ Hz to 10^6^ Hz) up to high frequency range (0.2 Hz to 20 GHz), and required support equipment such as an open ended coaxial line probe, an automated network analyzer for the high frequency signals and a spectrum analyzer for low frequency ones. The measurement method yielded 5% accuracy for dielectric constant (*ε*′) and 3% for dielectric loss (*ε*″). Like other methods presented here this capacitive method requires supporting equipment and is not suitable for outdoor testing. In the prevailing research works from the literature, the inductive concept has not been proposed and attempted for maturity determination of FFBs [20]. The proposed inductive concept is a non destructive testing method and show potentiality for outdoor testing [21,22]. The detection concept is based on the moisture content of the fruitlet, where the permeability value of water is 1.2566270 × 10^−6^. With a low permeability value compared to other materials especially metals, a high frequency range is used to assist the detection. Theoretically, it is expected that the value of inductance for unripe fruitlets will be higher than that of ripe fruitlets due to the moisture content of the fruitlet. In this paper, the investigation of an oil palm fruit sensor based on a novel resonant frequency technique is presented. The study approach involves the use of inductance values in the high frequency range that are used in determining the maturity of the oil palm FFBs, specifically the ripe and unripe fruitlets. Further research on the categories of maturity such as under-ripe and overripe categories are to be investigated and will be reported in our future works. Since the value of inductance measured is very small (on the order of μH), therefore resonant frequency is used for analysis in this investigation. The investigations on the frequency characteristics of the air coils of the sensor are studied to observe the effects of coil diameter as a preliminary evaluation. As for the inductance characteristics, the effect of the coil diameter on differences between samples non-distinguishable with the increase of the coil diameter value was studied. The effects of the variations in the coil diameter yields a significant difference of 0.02643 between unripe samples to air and 0.01084 for ripe samples to air. Results from this study would be useful in designing an oil palm fruit sensor based on the inductive concept and enhancing the potential of the sensor in classifying oil palm fruit maturity.

## 2. Oil Palm Fresh Fruit Bunches

### 2.1. Grading Standard by MPOB

Table 1 shows the conventional grading standard used by the Malaysian Palm Oil Board (MPOB), Sime Darby Palm Oil Mill standard and other FFB mill graders [15,20]. Malaysian harvesters mainly use the grading standard shown in Table 1. Apart from the fruitlet surface colour, the number of empty fruitlet sockets increases the accuracy of the maturity classification. The optimum age for the oil palm to harvest is after the 15th week after anthesis (WAA). There are two methods for expressing the number of loose fruit sockets in determining the maturity of oil palm FFBs.

**Figure 1.**
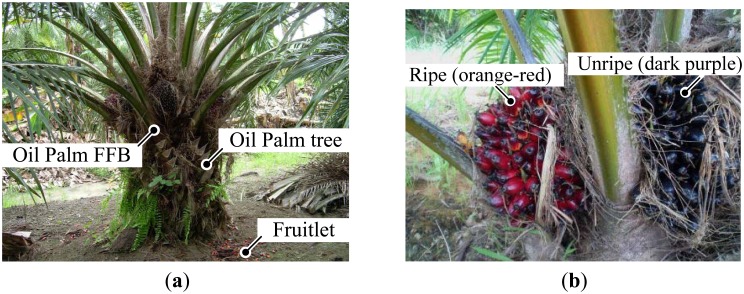
Samples of oil palm fruitlet (**a**) Oil palm fruit tree (**b**) Two category of samples.

One method is the number of loose fruits on the ground before the oil palm FFB is harvested and the other method is by the number of loose fruit sockets on the bunch, as shown in Figure 1.

### 2.2. Chemistry of Oil Palm

**Figure 2.**
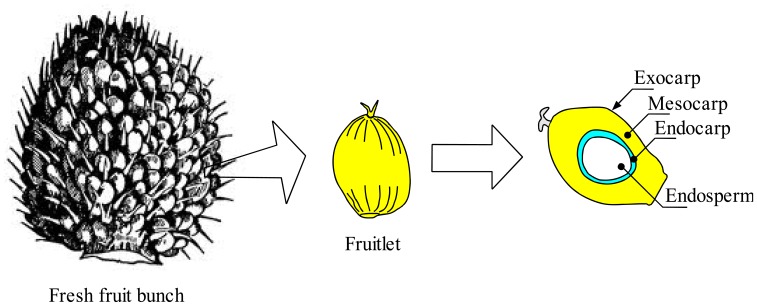
Ladder stages of the Oil Palm Fresh Fruit Bunch (FFB).

Determination of oil palm FBB depends highly on the number of loose fruits from the bunch and the surface color of the mesocarp. Generally, the ripening process starts from an apical fruitlet and gradually spreads to the basal part of the bunch [21] as shown in Figure 2.

The surface color of the mesocarp is highly affected by the amount of chlorophyll and triacylglycerols [22]. As the fruitlet grows, maximum accumulations of oil components known as triacylglycerols replace the chlorophyll in the mesocarp. Chlorophyll plays an important role in synthesizing carbohydrates in the fruitlet. The unripe fruit developed in the seventh WAA where the chlorophyll started to builds up. The maturity process of the fruitlet started from 7th WAA until the 15th WAA [22]. The ripening process of the fruitlet is assisted by the increased fruit photosynthesis supported by the amount of chlorophyll in the fruitlet. As the ripening process progresses, the amount of chlorophyll starts to degrade and the amount of triacylglycerols (TG) synthesized begins to increase rapidly. Table 2 summarizes the major constituents of the unripe and ripe fuitlets. The surface color of the immature fruitlet changes from black to purple and then from orange to red as the fruitlet ripens.

**Figure 3.**
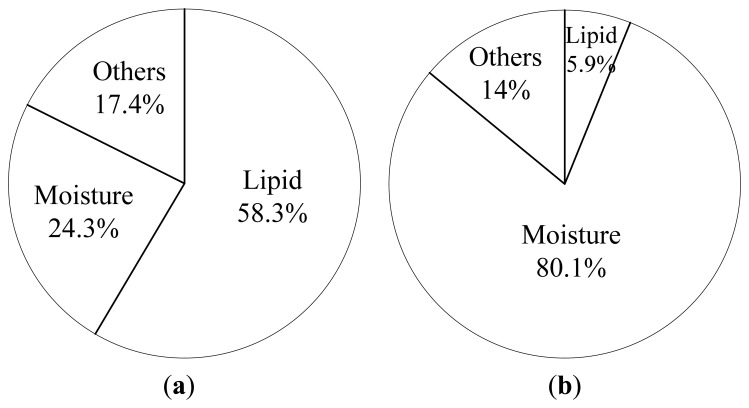
Chemical contents of the oil palm fresh fruit bunch (FFB) (**a**) Ripe (**b**) Unripe.

Figure 3 shows the chemical contents of the ripe and unripe fruitlet. The analysis of the chemical content is performed at the Food Technology Department, General Industrial Technology Center, Nagano Prefecture, Japan. More than 58% of the ripe fruitlet consists of lipid (oil) and 80% of the unripe fruitlet contains moisture (water). The remaining content of the oil palm fruit other than moisture and lipid (oil) is the fiber component of the fruit.

## 3. Materials and Methods

### 3.1. Introduction

The proposed inductive oil palm fruit sensor concept is based on the moisture content of the fruitlet. In view of the fact that the oil palm fruitlet is a non-conducting material, the permeability value of the fruitlet is very small. With a low permeability value compared to other materials, especially metals, a high frequency range is used to assist the detection. However, promising detection is shown at the resonant frequency of the air coil. Mathematically, the resonant frequency is given by Equation (1):

(1) $$f_{r}=\frac{1}{\sqrt{LC}}$$

where *f*_r_ and *L* are the resonant frequency and the inductance respectively. From Equation (1), it is observed that the resonant frequency is inversely proportional to the value of inductance if the value of capacitance is constant. As the value of inductance has a high impact on the resonant frequency, mathematically it can be computed from Equation (2):

(2) $$L=\frac{N^{2}\mu_{0}\mu_{r}A}{l}$$

where *N*, *μ*_0_, *μ*_r_, *A* and *l* are the number of turns, the permeability of free space, the permeability of material, the cross sectional area and length of the wire, respectively. Based on Equation (2), the value of inductance is highly dependent on the value of *μ*_r_ if other parameters are constant. It is known that the permeability value of water is 1.2566270 × 10^−6^ and the permeability of air is equivalent to *μ*_0_ which is 1.256637061 × 10^−6^. Based on the permeability value of water and air, it is expected that the value of measured inductance for unripe fruitlets will be higher compared to ripe fruitlets due to the percentage of moisture content in the fruitlets. From the experimental results, it is expected that the permeability value for oil palm is between the permeability of water and air.

### 3.2. Oil Palm Fruit Sensor

**Figure 4.**
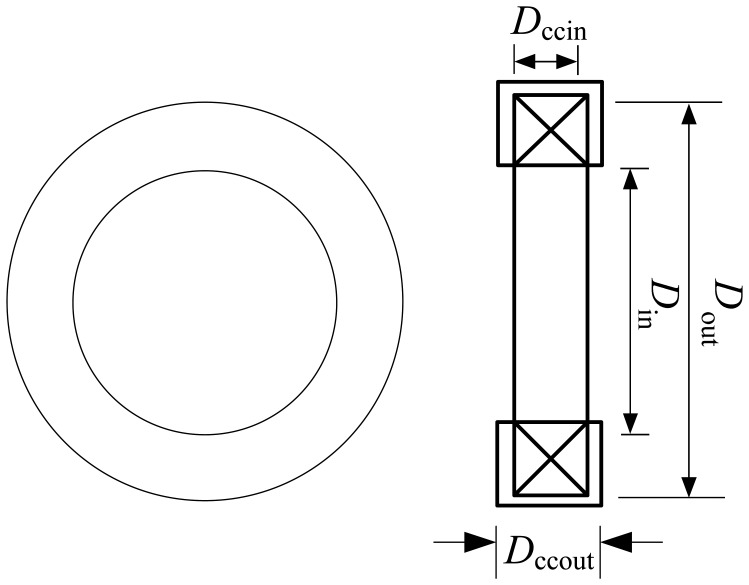
Structure of the air coil.

The Oil Palm Fruit Sensor consists of the air coil, air coil holder, a stand base and a U-shape yoke. Delrin^®^ as a non conducting material is chosen as material for fabrication of the sensor in order to minimize the flux disturbance in the sensor. Delrin^®^ is a crystalline plastic which possesses high tensile strength, creep resistance and toughness and exhibits low moisture absorption. It is chemically resistant to hydrocarbons, solvents and neutral chemicals [23]. The structure of the air coil is accompanied with a round shape coil casing and is hollow in the middle part as shown in Figure 4. The diameter of inner part of the sensor casing, *D_CC_*_in_ (hollow part) varies between 24 mm to 28 mm so that it can adapt to various sizes of oil palm fruitlets. The coil casing (*D_CC_*_out_) offers an approximately 2 mm air gap between the fruitlet and the air coil. A 0.25 mm diameter of copper wire is wound around the air coil with different turns of five, ten, fifteen and twenty turns. Samples of oil palm fruitlets are placed in the middle of the air coil. A U-shape yoke is designed to hold the sample in place and to avoid movement during the measurement stage. A stand base with multiple choices of distance to hold the U-shape yoke is designed to hold the sensor. Sixteen samples from two different categories are tested and plucked on the same day of the testing. Table 3 summarizes the structural dimensions of the air coil used in the frequency characteristics testing. Inductance and resonant characteristics are observed to evaluate the test measurements. The effect of the variations in the coil diameter in the resonant characteristics is evaluated.

### 3.3. Experimental Setup

**Figure 5.**
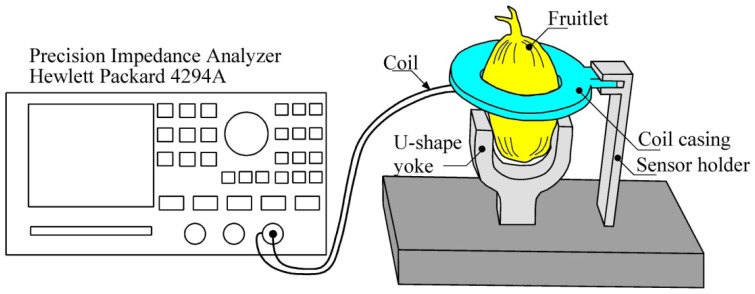
Frequency characteristics experimental setup.

A series of experiments are conducted to study the frequency characteristics of the air coil for the oil palm fruit sensor with the measurement setup. The sensor consists of the air coil, the sensor holder and the fruitlet holder as shown in Figure 5. The fruitlet holder is designed to hold the samples in place. This arrangement is necessary to minimize the errors due to disturbance to the sensor and the position of the fruitlets. The system consists of the sensor connected to a Hewlett Packard 4294A 40 Hz–100 MHz precision impedance analyzer. The frequency of the impedance analyzer is varied from 100 Hz to 100 MHz in steps of 10 Hz and the two categories each from the ten samples are tested. The distance between the sensor and the point of the coil at the impedance analyzer is fixed to minimize disturbances to the air coil when it is operated at higher frequency. The experiment started with the testing of the air coil of the sensor without samples. It is necessary to measure the self inductance of the air coil before all samples are tested. The experiment is conducted by running the sensor without sample up to 100 MHz frequency. The self inductance of the air coil is determined when the resonant frequency of the coil is reached. Then, the experiment continues with the samples from each category being tested until the frequency of the impedance analyzer reached 100 MHz. The resonant frequency of the air coil is observed for each sample from the category. Ten samples from two categories namely the unripe fruitlets and the ripe fruitlets are used every time the experiment is conducted. Table 4 summarizes the specifications for the frequency characteristics of the experimental setup. The specifications are set and standardized for the whole series of the experiment for all the ten samples.

### 3.4. Oil Palm Fruitlet Samples

Seven experimental sessions were conducted, and in each session, five samples of ripe and unripe fruitlets were used, respectively. For each sample, five readings were taken to ensure the repeatability of the measurements, however, only 16 samples out of 35 samples have been presented in this paper. The category is selected based on the surface colour of the fruitlets. The unripe fruitlets are dark purple in colour while the ripe fruitlets are orange in colour. For the unripe fruitlets, the samples are selected during the 7th week after anthesis (WAA) and for the ripe fruitlets on the 15th week after anthesis (WAA). The size of the fruitlets is chosen randomly based on the diameter of the air coil for the sensor. Each sample is selected and plucked from the same oil palm FFB on the day of testing. This is to ensure that the samples used are fresh and free from physical contamination.

## 4. Results

### 4.1. Coil Inductance Characteristics

**Figure 6.**
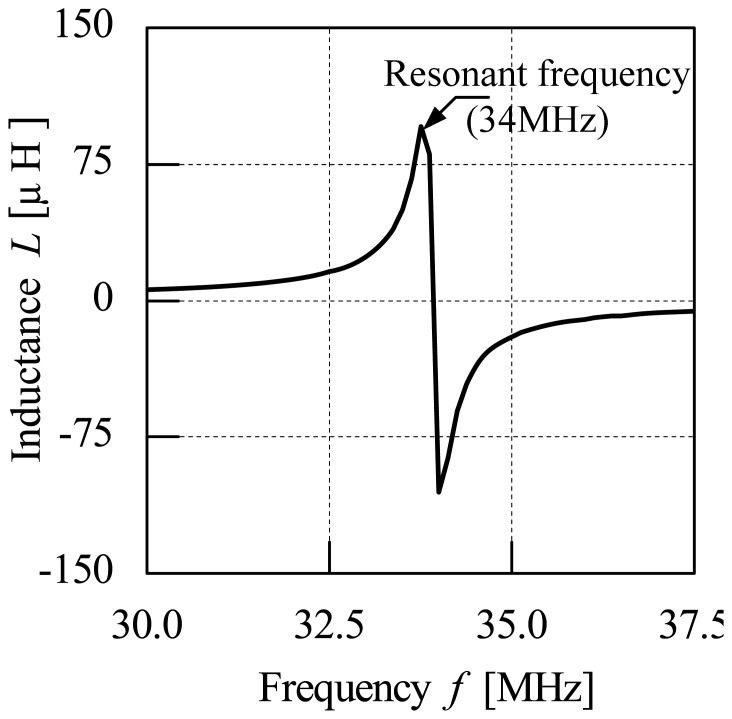
Inductance characteristics for the air coil.

The evaluation on the frequency characteristic of the air coil started with the inductance characteristics for each air coil. The inductance characteristics are observed at the resonant frequency of the air coil. Figure 6 shows the inductance characteristics of the air coil. The value of inductance for all types of air coil measured is very small (on the order of μH). The inductance characteristic is uniform for all types of air coil and is of similar nature. The inductance characteristics for all types of samples tested show similar patterns for all air coils. The patterns started with the inductance value increment and shoots to the maximum value at the resonant frequency. The value of inductance then decreases abruptly to a negative spike before returning to its initial state. The inductance characteristics each of the air coil type used in the frequency characteristics testing show similar trends in their characteristics.

### 4.2. Resonant Characteristics

**Figure 7.**
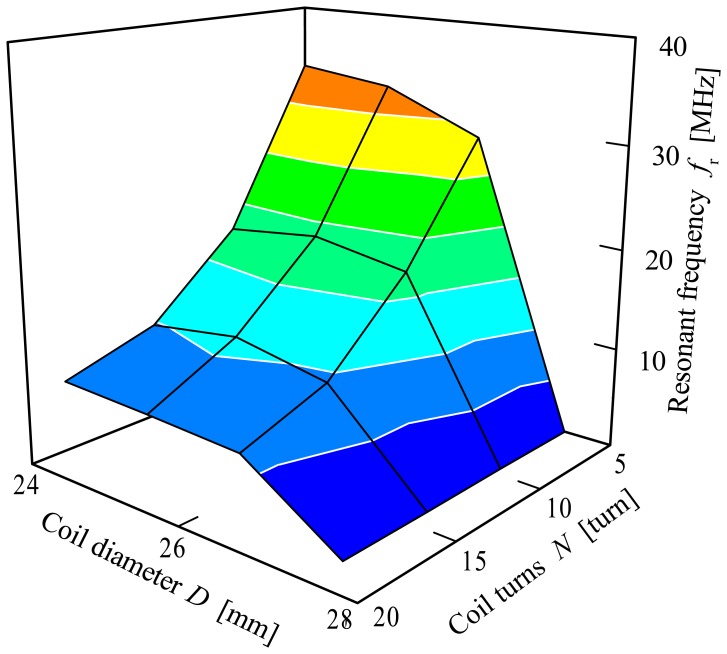
Frequency characteristics for the air coil.

Frequency characteristics of the air coil are concentrated on the inductance characteristics for each air coil, the effects of the coil's diameter of the inductance value as well as the resonant frequency of the air coil. From Figure 7, it can be seen that the resonant frequency of the air coil decreases as the coils diameter increases. The resonant frequency for the air coil decreases from 37.5 MHz to less than 10 MHz for variations of coil diameter range. Like the coil diameter, the coil turns have a similar effect on the resonant frequency of the air coil. The resonant frequency decreases as the number of coil turns decreases.

### 4.3. Inductance Characteristics of Ripe-Unripe Fruitlets for Different Coil Diameters

**Figure 8.**
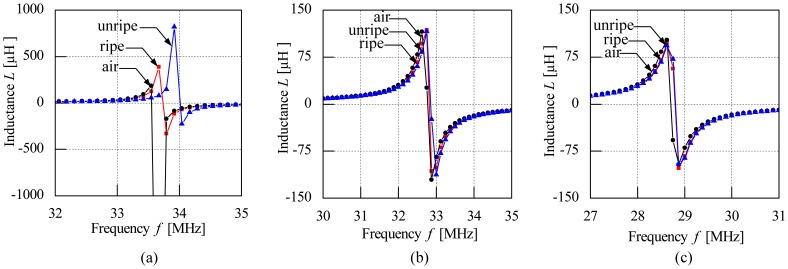
Inductance characteristics for different coil diameter (**a**) 24 mm (**b**) 26 mm (**c**) 28 mm.

The frequency characteristic of the air coil looked at the effects of the coil diameter on the inductance value and resonant frequency of the air coil. Three types of coil diameter were used in the experiments: 24 mm, 26 mm and 28 mm. However, the number of coil turns for each coil diameter is fixed at five times as the results from this number of coil turns produced distinct differences. Results show that the unripe fruitlets produce the highest inductance value as compared to ripe fruitlets for all types of air coil. The effects of the coil diameter are clearly seen when 24 mm air coil is used and the coil's diameter varies. The value of the resonant frequency showed a significant difference between samples as shown in Figure 8. However, the significant difference between the resonant frequency of the air coil between samples is minimized as the coil diameter is increased. The 24 mm diameter air coil show promising results as compared to the 26 mm and 28 mm air coil diameters, as the gap between ripe-unripe fruitlets is almost overlapped and hardly seen for 26 and 28 mm air coil diameters.

### 4.4. Effect of Coil Diameter on Ripe-Unripe Fruitlets

**Figure 9.**
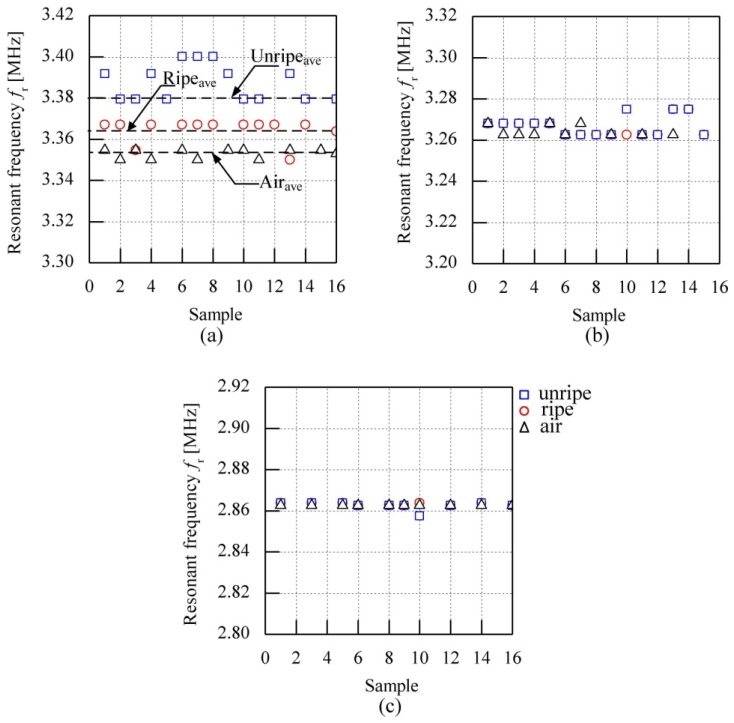
Effects of coil diameter (**a**) 24mm (**b**) 26mm (**c**) 28mm.

The resonant frequency is evaluated with a plot against sixteen samples throughout all sessions as shown in Figure 9. The value of the resonant frequency for all air coils from different samples is plotted against number of samples to observe the effects of coil diameter on the value of the resonant frequency of the air coil. The average value is calculated from sixteen samples and the percentage of gap between ripe to air and unripe to air is based on the average value. The calculated average values for air, unripe and ripe fruitlets are 3.35295, 3.37938 and 3.36379 MHz, respectively for a 24 mm air coil diameter. From Figure 9(a), the coil diameter at 24 mm shows a significant difference of 0.02643 MHz between unripe samples to air and 0.01084 MHz for ripe samples to air. As the coil diameter rises, the resonant frequency of the air coil decreases and the significant difference between samples is non-distinguishable. Table 5 summarizes the calculated average values for all types of sample and the differences between ripe to air and unripe to air for the 24 mm air coil diameter.

## 5. Conclusions

This paper presents the frequency characteristics of the air coil for an Oil Palm Fruit sensor based on the inductive concept. Two characteristics are observed in this study; the resonant characteristic as well as inductance characteristics of the air coil. For finding the resonant characteristics the effect of coil diameter is evaluated. The inductance characteristics showed a uniform pattern for all types of air coil throughout the whole series of the testing. As for effects of coil diameter, distinct differences between samples are clearly seen for the 24 mm coil diameter when the number of coil turns is fixed at five. The resonant frequency of the air coil is reduced as the coil's diameter rises. As for the inductance characteristics, the effect of the coil diameter with a distinct difference between samples are non distinguishable as the value of the coil diameter increases. The effect of coil diameter yields a 0.02643 MHz significant difference between unripe sample to air and 0.01084 MHz for ripe sample to air. Thus, this initial study would help improve the design of the air coil sensor to enhance the potential of the inductive coil resonant frequency technique for determining the maturity of oil palm fruitlets as well as of oil palm fresh fruit bunches to maximize the production of palm oil. A few other considerations for the sensor's design such as number of turns, diameter of the copper wire and shape of the air coil as well as sample preparation are to be further investigated to optimize the performance of the sensor.

## Figures and Tables

**Table 1.** Grading standard used by MPOB [17].

**Grading Method**	**Total Number of Empty Fruitlet Sockets**	**Mesocarp Colour**		
		**Yellow**	**Yellowish/Orange**	**Orange**
Number of loose fruit sockets on the bunch	0 0–10 >10	Unripe Unripe Unripe	Unripe Under-ripe Ripe	Ripe Ripe Ripe
Number of loose fruits on the ground	ripe over-ripe under-ripe	10%–50% of fruits detached from bunch 50%–90% of fruits detached from bunch 1–9 fruits detached from bunch		

**Table 2.** Characteristics of oil palm fruitlet.

**Characteristic**	**Unripe fruitlet**	**Ripe fruitlet**
Constituents	chlorophyll, sterols	carotenoids, triacylglycerols
Age	<12 WAA	16 WAA–20 WAA
Colour	Dark purple	Orange-red

**Table 3.** Structure dimensions of the air coil.

**Parameter/Part**	**Value/Type**
Outer diameter of air coil, *D*_out_ (mm)	Based on the number of coil turns
Inner diameter of air coil, *D*_in_ (mm)	26, 28, 30
Outer diameter of coil casing, *D_CC_*_out_ (mm)	40
Inner diameter of coil casing, *D_CC_*_in_ (mm)	24, 26, 28
Diameter of the copper wire (mm)	0.25

**Table 4.** Specifications for the frequency characteristics experimental setup.

**Parameter/Part**	**Value/Type**
Type of measurement setup	Series (*L*_s_ – *R*_s_)
Voltage (V)	0.5
Frequency (Hz)	100–100 × 10^6^
Sweep (points)	801

**Table 5.** Average value and difference between sample to air in coil diameter effect.

**Sample**	**Average Value (Mhz)**	**Differences between Sample to Air (Mhz)**	**Standard Deviation (Mhz)**
Air	3.35295	-	0.022
Ripe	3.36379	0.01084	0.055
Unripe	3.37938	0.02643	0.090
